# Effect of delayed graft function, acute rejection and chronic allograft dysfunction on kidney allograft telomere length in patients after transplantation: a prospective cohort study

**DOI:** 10.1186/s12882-015-0014-8

**Published:** 2015-02-18

**Authors:** Leszek Domański, Karolina Kłoda, Ewa Kwiatkowska, Ewa Borowiecka, Krzysztof Safranow, Arleta Drozd, Andrzej Ciechanowicz, Kazimierz Ciechanowski

**Affiliations:** Clinical Department of Nephrology, Transplantology and Internal Medicine, Pomeranian Medical University in Szczecin, Ul. Powstancow Wlkp. 72, 70-111, Szczecin, Poland; Department of Biochemistry and Human Nutrition, Pomeranian Medical University in Szczecin, Szczecin, Poland; Department of Biochemistry and Medical Chemistry, Pomeranian Medical University in Szczecin, Szczecin, Poland; Department of Laboratory Diagnostics and Molecular Medicine, Szczecin, Poland

**Keywords:** Ageing, AR, Cellular senescence, CAD, DGF, Telomere length

## Abstract

**Background:**

The outcome of kidney allograft transplantation is associated with numerous donor-dependent and recipient-dependent immunological and non-immunological factors. Studies on genetic factors affecting the non-immunological aspects, like ageing of the kidney allograft and patient outcome are still lacking. The aim of this study was the analysis of relative telomere length (RTL; T/S ratio) in the biopsy specimens of the transplanted kidney allograft and its correlation with the delayed graft function (DGF), acute rejection (AR) and chronic allograft dysfunction (CAD).

**Methods:**

The study enrolled 119 Caucasian kidney allograft recipients (64 M/55 F, mean age 47.32 ± 14.03; transplantation performed between 2001 and 2012). Organs were harvested from cadaveric donors (59.8 M/40.2 F, mean age 45.99 ± 14.62).

**Results:**

There were significant differences in RTL assessed in kidney allograft biopsy specimens collected 3–6 months after transplantation between patients with DGF and without DGF (181.8 ± 82.0 vs. 284.6 ± 149.6; p < 0.05) and in RTL of kidney allograft biopsy specimens collected 18–60 months after transplantation between patients with AR and without AR (188.1 ± 162.1 vs. 263.3 ± 134.7; p = 0.047). There were significant differences in RTL assessed in kidney allograft biopsy specimens collected 12–24 months after transplantation between patients with CAD and without CAD (168.0 ± 120.0 vs. 282.1 ± 158.4; p = 0.038).

**Conclusions:**

Duration of dialysis before transplantation and PRA influence the kidney allograft ageing. Telomere length assessed in biopsy specimens collected in the peri-transplant period predicts the long-term kidney allograft function. Complications of kidney transplantation, like DGF, AR and CAD are linked with the telomere length and thus, graft ageing.

## Background

The outcome of kidney allograft transplantation is associated with numerous donor-dependent and recipient-dependent immunological and non-immunological factors [1-3]. Among them, genetic factors are of great importance for restoring and maintaining kidney function and determining the possibility of acute (AR) and chronic rejection resulting in chronic allograft dysfunction (CAD) [4-6]. A series of reports regarding polymorphisms of genes implicated in the immune response after transplantation have been published [7-9]. However, studies on genetic factors affecting the non-immunological aspects, like ageing of the kidney allograft and patient outcomes are still lacking. Hence, there is growing interest in cellular senescence and an increasing number of attempts to associate telomere length of the transplanted organ cells with its survival [10].

Telomeres are located at the ends of eukaryotic chromosomes and their functional role is protection against the degradation of chromosomes and the maintenance of genome integrity and stability. They are comprised of tandem nucleotides repeats (TTAGGG) [11]. The protective activity of the telomeres depends on many factors – proteins linked with their function (TNF receptor associated factor 1 and 2 – TRAF1, TRAF2, Ku86), the level of telomerase activity and the telomere length itself. Telomere shortening of about 50–200 bp occurs with each cell division in the absence of active forms of telomerase. When the critical telomere length is reached, the cell starts the process of apoptosis. If the cell has the ability to regenerate the telomeres, it becomes immortal and divides without control. Another possibility is that after using the replication potential, the cell may also stay in the G1 phase not responding to external stimuli, yet retaining metabolic activity and contributing to the formation of inflammatory process [12].

The association between many types of cancer and non-cancer diseases (arterial hypertension, cardiovascular disease) and telomere length, as well as telomerase activity, has been confirmed [13-15]. Telomere length was also correlated with the deterioration of kidney function and ageing of the kidney. Moreover, differences in telomere length between the renal medulla and the cortex were observed [16,17]. It is considered that chronic rejection of a kidney allograft may be associated with the increased ageing of the transplanted organ. A significant loss of telomere length, not related to cell division, has been observed before the kidney rejection process [18]. Therefore, the aim of this study was analysis of the relative telomere length (RTL; T/S ratio) in biopsy specimens of the transplanted kidney allograft and its correlation with delayed graft function (DGF), AR and CAD.

## Methods

Design: Prospective cohort study.

Setting: Clinical Department of Nephrology, Transplantology and Internal Medicine and Transplant Outpatient Clinic

### Participants

The study enrolled 119 Caucasian kidney allograft recipients (64 M/55 F, mean age 47.32 ± 14.03; transplantation performed between 2001 and 2012). Organs were harvested from cadaveric donors (59.8 M/40.2 F, mean age 45.99 ± 14.62). The population of recipients was dived into subgroup A – up to 2 years after transplantation (n = 70), and subgroup B – more than 2 years after transplantation (n = 49). The division was necessary to set the inclusion criteria. Subgroup A included first renal allograft recipients recruited consecutively immediately after transplantation and after giving their consent to participate in the study, while subgroup B included the first renal allograft recipients with a functioning organ, recruited from the Transplant Outpatient Clinic that delivers ongoing care for kidney transplant recipients following discharge, after giving their consent to participate in the study. Exclusion criteria were: more than 1 renal transplantation, lack of consent, or organ loss/return to dialysis. Kidney allograft recipients’ detailed characteristics are presented in Table 1. The main causes of impaired kidney function before transplantation were glomerulonephritis, type 1 and 2 diabetes (T1DM and T2DM), arterial hypertension and autosomal dominant polycystic kidney disease (ADPKD). Arterial hypertension, post-transplant diabetes (PTDM) and atherosclerosis were the main comorbid conditions.

**Table 1:** **Clinical characteristics of the study group**

	**Recipients (n)**	**Mean ± SD or %**
Mean age [years]	119	47.32 ± 14.03
Sex	119	53.8 M%
BMI [kg/m^2^]	67	25.75 ± 4.50
AH	115	79.13%
HD before Tx	84	78.6 %
Preemptive Tx	84	4.76%
Mean time of D before Tx [months]	80	28.02 ± 19.65
CIT [hours]	93	17.7 ± 6.65
DGF frequency	119	30.25%
AR frequency	119	28.57%
CAD frequency	119	14.29%
Mortality	119	2.52%

SD – standard deviation, M- males, AH – arterial hypertension, HD - hemodialysis, Tx – transplantation, D – dialysis, CIT – cold ischemia time, DGF – delayed graft function, AR – acute rejection, CAD – chronic allograft dysfunction.

The following parameters were recorded: the recipient and donor ages and gender, recipient’s body mass index (BMI), the type of dialysis treatment and its duration before transplantation, cause of impaired kidney function, residual diuresis, panel reactive antibodies (PRA), cold ischaemia time (CIT), date of the transplantation, comorbid conditions, occurrence and type of infection after transplantation. The frequencies of DGF, AR and CAD were observed. The diagnosis of DGF was determined as the need for dialysis during the first 7 days after transplantation. Identification of AR was confirmed clinically (pain and/or swelling of the kidney graft, body temperature ≥38°C, elevated serum creatinine ≥25% in the absence of other pathology including infection, urinary tract obstruction, allograft artery stenosis, or cyclosporine and tacrolimus toxicity), and by biopsy review. The diagnosis of CAD was based on functional and morphological (biopsy confirmed) deterioration of a renal allograft at least 3–6 months after transplantation. Blood samples were collected for creatinine concentration evaluation during the first 7 days after transplantation, and 1, 3, 6, 12, 18, 24, 30, 36, 48 and 60 months after kidney transplantation. Creatinine concentration assessment was performed using a colorimetric method. Biopsy specimens were collected for analysis of the RTL and renal pathologist review (Banff working classification criteria were used) in the peri-transplant period (biopsy ‘0’), 3, 6, 12, 18, 24, 36, 48 and 60 months after transplantation and in the case of deteriorating renal transplant function. All patients received standard immunosuppressive protocol with triple drug therapy including calcineurin inhibitor (tacrolimus), mycophenolate mofetil or mycophenolate sodium, and steroids. Some individuals in subgroup B received cyclosporine, azathioprine and steroids after transplantation, but were then converted to tacrolimus and mycophenolate mofetil at least 6 years prior to the study. If necessary, the immunosuppressive protocol was modified. Informed consent was obtained from all patients. The local ethics committee of the Pomeranian Medical University in Szczecin, Poland, approved protocol of the study.

### Relative telomere length analysis

DNA was extracted using a mini column-based DNA isolation kit (A&A Biotechnology, Gdynia, Poland) and stored at −20°C. A high concentration genomic DNA sample was prepared in decimal concentrations to cover all possible measurements. According to standard procedure, telomere length was assessed using two pairs of primers, i.e. telomere-specific and a single copy gene-specific (albumin). The design of primers specific to a difficult sequence of telomeres is challenging and there are already numerous protocols for this application [19,20]. We used the primers that had already been shown to work specifically [19], as we found them to be the most optimal of all of the primers tested in the context of specificity and to result in no primer-dimer or non-specific products.

The PCR conditions used to amplify the telomere fragment were as follows: Initial denaturation and polymerase activation (hot start) was performed in 95°C for 10 min followed by two cycles of 94°C/15 s and 49°C/15 s without fluorescence acquisition. The signal was detected during another 40 cycles i.e. 94°C/10 s, 66°C/10 s and 72°C/10 s. Melting analysis (65-95°C range, 0.2°C resolution) at the end of the reaction was performed to verify the specificity of the product and indicated Tm = 81.7. The efficiency of the reaction was no lower than 97.8%. Importantly, this result was repeatable for all of the samples that were analysed (in serial dilutions). After the optimisation of primer concentration, which was estimated to be 0.5 μM, we selected the optimal magnesium chloride concentration of 2.5 mM. Similarly, reaction conditions used to identify the optimal annealing temperature, magnesium chloride and primer concentrations for albumin were as follows: denaturation 95°C /10 min – hot start; followed by 45 cycles 94°C/10 s, 61°C/10 s and 72/10 s. The Tm of the product (analysis performed as above) was 80.7 and the efficiency was 99.6%. The concentration of primers was 0.5 μM and magnesium chloride 2.5 mM. The telomere length was assessed using a qPCR system (Roche, LC 2.0) and SybrGreen kit (Roche, Manheim, Germany).

### Statistical analysis

Since distributions of most quantitative variables were significantly different from normal distribution (p < 0.05, Shapiro-Wilk test), we used non-parametric tests. Spearman’s rank correlation coefficient was used to analyse correlations between variables and the Mann–Whitney U test was used to compare values between groups. General linear model (GLM) was used for multivariate linear regression analysis with logarithmically transformed RTL as dependent variable. Associations with a p-value <0.05 were considered statistically significant. Calculations were performed with Statistica 10 software.

## Results

Since, the sub-group B patients were recruited 2 years and later after transplantation, the 0–3 and 3–6 months time ranges regard sub-group A individuals only. Among the individuals with DGF, AR was observed in 9 and CAD in 6 patients. The number of 25 subjects without DGF underwent AR and 11 developed CAD in later time period. The analysis of RTL in kidney allograft biopsy specimens and different variables like recipients’ and donors’ age, duration of dialysis before transplantation, PRA and creatinine concentration revealed significant, positive and negative correlations, which are presented in detail in Table 2. There was a significant positive association between the age of recipients and RTL in biopsies performed 6 months after transplantation (p = 0.049). A significant negative association between the age of donors and RTL in biopsies performed 12–24 and 12–60 months after transplantation was observed (p = 0.017 and p = 0.045 respectively). Negative correlations between the longer dialysis duration before the transplantation and RTL in biopsy specimens collected 24 and 18–60 months after transplantation were observed (p = 0.024 and p = 0.05, respectively). There was a significant negative association between PRA and RTL in biopsies performed 6 months after transplantation (p = 0.04). There were strong associations between long-term creatinine concentrations (12, 12–18 and 18 months) and RTL in biopsy specimens collected in the short period after transplantation (p = 0.01, p = 0.009, p = 0.006, respectively). There were no associations between CMV infection and RTL in biopsy specimens collected at different time points after transplantation.

**Table 2:** **Overview of the statistically significant correlations between different variables and relative telomere biopsy specimens length**

**Correlation of the relative telomere biopsy length**	**N**	**Rs**	**p value**
Recipients’ age & RTL 6 months	12	+0.58	**0.049**
Donors’ age & RTL 12–24 months	43	−0.36	**0.017**
Donors’ age & RTL 12–60 months	53	−0.28	**0.045**
Duration of dialysis before Tx & RTL 24 months	9	−0.74	**0.024**
Duration of dialysis before Tx & RTL 18–60 months	17	−0.47	**0.05**
PRA & RTL 6 months	8	−0.73	**0.04**
Creatinine 12 months & RTL 0 months	17	−0.60	**0.01**
Creatinine 12–18 months & RTL 0 months	17	−0.61	**0.009**
Creatinine 18 months & RTL 0–6 months	24	−0.54	**0.006**

RTL – relative telomere length, BMI – body mass index, Tx – transplantation, PRA – panel reactive antibodies, p value calculated for Spearman’s rank correlation coefficient (Rs).

There were no differences in RTL assessed in biopsies performed 0–3, 12–24 and 18–60 months after transplantation between patients with DGF and without DGF. However, significant differences in RTL assessed in kidney allograft biopsy specimens collected 3–6 months after transplantation between patients with DGF and without DGF were observed (181.8 ± 82.0 vs. 284.6 ± 149.6; p < 0.05) (Table 3). There were no differences in RTL assessed in biopsies performed 0–3, 3–6 and 12–24 months after transplantation between patients with AR and without AR. However, significant differences were found in RTL assessed in kidney allograft biopsy specimens collected 18–60 months after transplantation between patients with AR and without AR (188.1 ± 162.1 vs. 263.3 ± 134.7; p = 0.047) (Table 4). In the case of sub-group A individuals (up to 2 years after transplantation) 3 of them did not recover the graft function after AR fully and developed CAD. There were no differences in RTL assessed in biopsies performed 0–3 and 3–6 months after transplantation between patients with CAD and without CAD. However, significant differences in RTL assessed in kidney allograft biopsy specimens collected 12–24 months after transplantation between patients with CAD and without CAD were observed (168.0 ± 120.0 vs. 282.1 ± 158.4; p = 0.038). The RTL in specimens collected 18–60 months after transplantation was shorter in patients with CAD; however, these differences were not statistically significant (Table 5). We have found in the multivariate linear regression analysis adjusted for donor’s and recipient’s age (Table 6) that DGF is an independent factor associated on the border of statistical significance with shorter RTL 3–6 months after transplantation (p = 0.089). In similar analyses we observed that AR is an independent factor significantly associated with shorter RTL 18–60 months after transplantation (p = 0.048) and CAD is an independent factor significantly associated with shorter RTL 12–60 months after transplantation (p = 0.02).

**Table 3:** **Mean values of the RTL in kidney allograft biopsy specimens collected from patients with DGF and without DGF**

**Biopsy [months]**	**Patients with DGF**		**Patients without DGF**		**p value**
	**Relative telomere length**		**Relative telomere length**		
	**N**	**Mean ± SD**	**N**	**Mean ± SD**	
Biopsy 0-3	15	234.2 ± 108.8	47	287.2 ± 146.0	0.25
Biopsy 3-6	9	181.8 ± 82.0	30	284.6 ± 149.6	**<0.05**
Biopsy 12-24	11	199.1 ± 105.8	34	278.8 ± 167.2	0.18
Biopsy 18-60	9	193.1 ± 105.3	25	246.4 ± 161.4	0.56

RTL – relative telomere length, DGF- delayed graft function, SD- standard deviation, p value calculated with the Mann–Whitney U test.

**Table 4:** **Mean values of the RTL in kidney allograft biopsy specimens collected from patients with AR and without AR**

**Biopsy [months]**	**Patients with AR**		**Patients without AR**		**p value**
	**Relative telomere length**		**Relative telomere length**		
	**N**	**Mean ± SD**	**N**	**Mean ± SD**	
Biopsy 0-3	10	270.8 ± 151.6	52	275.0 ± 138.0	0.99
Biopsy 3-6	8	253.2 ± 184.4	31	262.9 ± 133.8	0.64
Biopsy 12-24	18	241.7 ± 163.7	27	271.1 ± 154.7	0.44
Biopsy 18-60	14	188.1 ± 162.1	20	263.3 ± 134.7	**0.047**

RTL – relative telomere length, AR – acute rejection, SD- standard deviation, p value calculated with the Mann–Whitney U test.

**Table 5:** **Mean values of the RTL in kidney allograft biopsy specimens collected from patients with CAD and without CAD**

**Biopsy [months]**	**Patients with CAD**		**Patients without CAD**		**p value**
	**Relative telomere length**		**Relative telomere length**		
	**N**	**Mean ± SD**	**N**	**Mean ± SD**	
Biopsy 0-3	5	343.5 ± 79.6	57	268.3 ± 141.9	0.15
Biopsy 3-6	4	336.5 ± 91.4	35	252.3 ± 146.0	0.15
Biopsy 12-24	9	168.0 ± 120.0	36	282.1 ± 158.4	**0.038**
Biopsy 18-60	10	178.7 ± 132.8	24	254.6 ± 152.3	0.16

RTL – relative telomere length, CAD – chronic allograft dysfunction, SD- standard deviation, p value calculated with the Mann–Whitney U test.

**Table 6:** **Multivariate linear regression analysis adjusted for donor’s and recipient’s age of the independent risk factors associated with logarithmically transformed RTL as dependent variable**

**Risk factor**	**RTL 3–6 months**	**RTL 12–60 months**	**RTL 18–60 months**
	**p value**	**p value**	**p value**
DGF	0.089 *	NS	NS
AR	NS	NS	0.048*
CAD	NS	**0.02** *	NS

DGF – delayed graft function, AR – acute rejection, CAD – chronic allograft nephropathy, RTL – relative telomere length; p values calculated with general linear model.

*negative association (risk factor associated with lower RTL).

## Discussion

In the present study, we found that age of the recipient is positively significantly correlated with RTL and that age of the donor, duration of the dialysis before the transplantation, PRA and long-term creatinine concentrations are negatively significantly correlated with RTL in biopsy specimens collected at different time points. Moreover, we observed a significant shortening of the telomere length in patients with DGF, AR and CAD. In the study of Oetting et al., telomere length was determined in kidney allograft recipients and their donors. Significant associations were found between log-transformed telomere length and donor age (p = 3.8 × 10^−4^), as well as recipient age (p = 5.6 × 10^−8^). No associations between log-transformed telomere length and AR or CAD were found [10]. These results are partly in agreement with our observations (age correlation); however, the associations reported by Oetting et al. for telomere length with AR and CAD are contrary to our findings. The differences in the results can be easily explained; whereas Oetting et al. analysed the telomere length in peripheral blood white blood cells, we examined the telomere length of kidney allograft biopsy specimens.

The generally observed variations in telomere length were thought to be related to longevity potential. It was confirmed that individuals who inherit longer than average telomere length tend to have increased lifespans. Because of the strong association between chronological age and telomere length, its measurement has been proposed as a marker evaluation of biological age for tissues and organs, including kidney allografts [21-23]. Based on an observation that chronological donor age is the most potent predictor of long-term outcome after kidney transplantation, Koppelstaetter et al. hypothesised that an estimate of telomere length and mRNA expression levels of the cell cycle inhibitors in zero hour biopsies would be of high predictive value. The authors observed a negative significant correlation between RTL and donor age. Moreover, they found that RTL was significantly negatively associated with serum creatinine concentration measured 12 months after transplantation (p = 0.035). These observations are in strong agreement with our results. Unfortunately, Koppelstaetter et al. did not analyse the correlations between RTL and DGF, AR or CAD. They did however correlate other variables like gender, CIT, PRA and AR with creatinine concentration 12 months after transplantation and found no significant associations [24].

There has been a lack of studies assessing the telomere length in regard to immunological complications after kidney transplantation. A report by Joosten et al. linked the telomere shortening with CAD in rats. They found a significant reduction in telomere length in all transplants and suggested that one of the causes of telomere shortening may be ischaemia and reperfusion injury resulting in the production of reactive oxygen species. More importantly, these authors state that accelerated aging of the renal allograft is not only associated with the age of the donor or the recipient, but also with the damage to the transplanted organ directly affecting the telomere shortening [18,25]. We made similar observations in our study. Despite the influence of donor and recipient age on the RTL, post-transplant complications like DGF, AR and CAD significantly affected telomere shortening. Moreover, these effects were ‘time-related’. The frequency of DGF, which is the result of ischaemia and reperfusion injury, was significantly associated with RTL in biopsies specimens collected 3–6 months after transplantation. We found no such association in regard to specimens collected 0–3 months after transplantation, because some of the renal allografts had undergone biopsy ‘0’ during CIT, before reperfusion and thus were not exposed to the damage yet. Specimens collected 3–6 months after transplantation were originating from kidneys exposed to the ischemia-reperfusion injury resulting in RTL decrease. Moreover, we can assume that the effect of DGF on the telomere shortening sustained until 6 months after transplantation and was no longer present in later time period possibly due to normal telomerase activity. Gingell-Littlejohn et al. correlated the telomere length in pre-implantation time zero biopsies with renal allograft function up to 1 year after transplantation. They also assessed the CDKN2A ageing biomarker expression. The authors found a significant association between telomere length shortening and deteriorating eGFR 6 months and 1 year after transplantation; however, they did not correlate telomere length with DGF and AR [26]. We found a significant association between AR occurrence and telomere shortening in biopsy specimens collected 18–60 months after transplantation. The majority of AR episodes can be observed during the first post-transplant year and, in some cases, even later resulting in graft function impairment and fibrosis. The shorter RTL because of AR were observed in biopsies performed in the long-term period after transplantation, possibly due to damage related changes in telomerase activity.

## Conclusions

It is important to indicate modifiable factors like duration of dialysis before transplantation and PRA and their influence on the accelerated ageing of the transplanted organ in order to improve transplantation outcome. Telomere length assessed in biopsy specimens collected in the peri-transplant period predicts the long-term kidney allograft function. Complications of kidney transplantation, like DGF, AR and CAD are linked with the telomere length and thus, graft ageing.

## Abbreviations

ADPKD: Autosomal dominant polycystic kidney disease
AR: Acute rejection
BMI: Body mass index
CAD: Chronic allograft nephropathy
CIT: Cold ischemia time
DGF: Delayed graft function
F: Females
M: Males
PCR: Polymerase chain reaction
PRA: Panel reactive antibodies
PTDM: Post-transplant diabetes
RTL: Relative telomere length
TRAF1: TRAF2, TNF receptor associated factor 1 and 2
T1DM: T2DM, Type 1 and 2 diabetes
